# Recent Advances in Understanding the Influence of Zinc, Copper, and Manganese on the Gastrointestinal Environment of Pigs and Poultry

**DOI:** 10.3390/ani11051276

**Published:** 2021-04-29

**Authors:** Leon J. Broom, Alessandra Monteiro, Arturo Piñon

**Affiliations:** 1 Gut Health Consultancy, Exeter EX14 1QY, Devon, UK; guthealthconsultancy@gmail.com; 2 Faculty of Biological Sciences, University of Leeds, Leeds LS2 9JT, UK; 3 Animine, 10 Rue Léon Rey-Grange, 74960 Annecy, France; amonteiro@animine.eu

**Keywords:** copper, manganese, zinc, pig, poultry, gut health, intestine, trace mineral, microbiome

## Abstract

**Simple Summary:**

Pigs and poultry, similar to humans, need regular consumption of zinc, copper, and manganese for normal functioning. To ensure adequate dietary intake, and prevent deficiency, their diets are supplemented with sufficient, often excessive, levels of these minerals or even at higher levels, which have been associated with improvements in their health and/or growth. However, if provided in excess, mineral quantities beyond those required are simply excreted from the animal, which is associated with negative consequences for the environment and even the development of antimicrobial resistance. Therefore, it is of great interest to better understand the dynamics of zinc, copper, and manganese in the intestine of pigs and poultry following consumption of supplemented diets, and how the requirements and benefits related to these minerals can be optimized and negative impacts minimized. The intestine of pigs and poultry contains vast numbers of microorganisms, notably bacteria, that continually interact with, and influence, their host. This review explores the influence of zinc, copper, and manganese on these interactions and how novel forms of these minerals have the potential to maximize their delivery and benefits, while limiting any negative consequences.

**Abstract:**

Zinc, copper, and manganese are prominent essential trace (or micro) minerals, being required in small, but adequate, amounts by pigs and poultry for normal biological functioning. Feed is a source of trace minerals for pigs and poultry but variable bioavailability in typical feed ingredients means that supplementation with low-cost oxides and sulphates has become common practice. Such trace mineral supplementation often provides significant ‘safety margins’, while copper and zinc have been supplemented at supra-nutritional (or pharmacological) levels to improve health and/or growth performance. Regulatory mechanisms ensure that much of this oversupply is excreted by the host into the environment, which can be toxic to plants and microorganisms or promote antimicrobial resistance in microbes, and thus supplying trace minerals more precisely to pigs and poultry is necessary. The gastrointestinal tract is thus central to the maintenance of trace mineral homeostasis and the provision of supra-nutritional or pharmacological levels is associated with modification of the gut environment, such as the microbiome. This review, therefore, considers recent advances in understanding the influence of zinc, copper, and manganese on the gastrointestinal environment of pigs and poultry, including more novel, alternative sources seeking to maintain supra-nutritional benefits with minimal environmental impact.

## 1. Introduction

Zinc (Zn), copper (Cu), and manganese (Mn) are prominent essential trace (or micro) minerals, meaning they are required in small, but adequate, amounts by livestock for normal biological functioning. As drinking water is not generally considered a major source of minerals, feed and supplements are the primary mineral source for livestock [1]. However, due to potentially inadequate trace mineral contents and variations in bioavailability amongst typical feed ingredients, trace mineral supplementation, traditionally in the form of low-cost oxides and sulphates, invariably providing high safety margins, have become common practice [2,3]. Moreover, some trace minerals (e.g., Cu and Zn) have been provided at supra-nutritional or pharmacological levels to improve the health and/or performance of monogastric species. Given the fact that absorption of many minerals from the gastrointestinal tract is carefully regulated and efficiency decreases as the mineral intake increases [1], much of the oversupply of trace minerals is simply excreted into the environment, which can be toxic to plants and microorganisms or promote the microbial acquisition and dissemination of genes conferring resistance to antimicrobial agents [3,4]. As a consequence, there has been increasing appraisal of, and legislation relating to, more precisely supplying trace minerals to livestock [5].

The essentiality of Zn, Cu, and Mn for health was demonstrated in rats in the early 20th century. The history of essential trace mineral discovery and their functional importance has been expertly outlined by Suttle [1]. Briefly, Cu, as a component of numerous enzymes and proteins, is involved in cellular respiration, antioxidant activity, iron transport, pigmentation, and connective tissue development (Table 1). Manganese has been found to be necessary for normal skeletal and reproductive development, with enzymes involved in lipid and carbohydrate metabolism, antioxidant activity (such as Cu and Zn), and proteoglycan synthesis activated by the trace mineral. Meanwhile, the functions of Zn are extensive, with many enzymes (>300), transcription factors (>2000), and proteins involved in signaling and metabolic pathways requiring Zn.

The small intestine, particularly the duodenum, is considered the primary site for the absorption of these trace minerals. This absorption occurs via both specific (e.g., Cu or Zn transporter 1 (ZnT1)) and/or less specific transporters (e.g., divalent metal transporter 1 (DMT1) in the intestine, which can be regulated by a host biological requirement and influenced by digestive dynamics [1]. Absorption of trace mineral ions can, for example, be affected by a competition for transport systems, host specific trace mineral status, the presence of phytate that can complex mineral ions, and/or gastric acid production and thus their degree of solubilization [6]. In addition, the form of the trace mineral (e.g., inorganic vs. organically-complexed) can influence these interactions and its bioavailability [2]. Moreover, feeding supra-nutritional or pharmacological levels of a trace mineral could lead to mineral imbalances and deficiencies [7].

Excretion of Zn, Cu, and Mn occurs primarily via the gastrointestinal tract, with minimal excretion via urine. Collectively, Zn, Cu, and Mn homeostasis is principally achieved through modulation of their absorption from, as well as excretion into, the gastrointestinal tract (GIT) [8]. In addition, concerns regarding the appropriateness of older recommendations for modern livestock genotypes under current rearing practices, and/or the commercial use of high safety margins or supra-nutritional levels, put the GIT environment at the center of host-mineral interactions.

**Table 1:** Some important functions of Zn, Cu, and Mn in livestock and associated dietary recommendations.

Trace Mineral	Functions	Example of NRC [9,10] Recommendations	Example of Commercial Specifications (average)	Reference (s)
Zinc	Fat absorption (phospholipase A2) Antioxidant activity (superoxide dismutase) Hydrolysis of phosphate esters (alkaline phosphatase) Formation of carbon dioxide (carbonic anhydrase)	Growing pigs 50–100 mg/kg	Up to 534 mg/kg	[11]
		Broiler chickens 40 mg/kg	110 mg/kg	[12]
Copper	Copper transport (caeruloplasmin) Cellular respiration (cytochrome oxidase) Antioxidant activity (superoxide dismutase) Iron absorption and transport (hephaestin, caeruloplasmin) Pigmentation (tyrosinase) Connective tissue (lysyl oxidases)	Growing pigs 3–6 mg/kg	Up to 82.5 mg/kg	[11]
		Broiler chickens 5–8 mg/kg	16 mg/kg	[12]
Manganese	Lipid and carbohydrate metabolism (pyruvate carboxylase) Antioxidant activity (superoxide dismutase) Proteoglycan synthesis (e.g., cartilage) (glycosyltransferase)	Growing pigs 2–4 mg/kg	Up to 70.3 mg/kg	[11]
		Broiler chickens 60 mg/kg	120 mg/kg	[12]

## 2. Zinc, Copper, and Manganese Influences on Intestinal Health

As essential nutrients, trace minerals are necessary for important cellular and biological processes. However, in excess, essential trace minerals are harmful to organisms and can have an antimicrobial activity, notably Cu and Zn [13,14,15]. Therefore, the dynamic interplay between the host, the microbiome, and these trace minerals help shape the GIT environment.

### 2.1. Zinc in Pigs and Poultry

Zinc is supplemented into the diet of swine to meet requirements and prevent deficiency, while, in recent decades, much higher inclusions of Zn (i.e., 2000–3000 mg Zn/kg) (as ZnO) became common practice for weaned piglets to reduce the incidence of diarrhea and to promote growth performance. While the precise mode(s) of action behind the supranational benefits are debated, evidence, including diarrhea reduction, point towards effects on the gut environment, including the composition and activity of the microbiota. There is also interest in understanding the GIT effects of supplemental Zn at levels designed to more closely meet the nutritional requirements of pigs, particularly in light of the pressures to reduce Zn excretion and associations with antimicrobial resistance.

Various studies have reported that pharmacological dietary inclusions of ZnO modify the GIT microbial community, including reductions in diversity and evenness [16], anaerobes [17], *Enterobacteriaceae* [18], clostridia and coliforms [19], *E. coli* [20], lactic acid bacteria and lactobacilli [21], and/or increases in coliforms and enterococci [21] or lactobacilli [19], evidently with opposite or contradictory effects sometimes reported. More recently, Pieper et al. [16] reported higher abundances of *Bacteroides*, *Parabacteroides*, *Collinsella*, *Acetivibrio*, *Blautia*, *Coprococcus*, *Faecalibacterium*, *Subdoligranulum* and lower abundances of *Megasphaera*, *Dialister*, *Acidaminococcus*, and *Ruminococcus* in the colon of weaned pigs fed 2500 mg/kg ZnO using metagenomic sequencing. Significant or similar effects on the gut microbiota have been reported using alternative sources of Zn, often at much lower levels of supplementation. For example, a potentiated ZnO source (110 mg/kg feed) had similar effects on the ileal microbiota as 2400 mg/kg Zn from conventional ZnO [20], while Zn-Lysinate (110 mg/kg) clustered separately from both Zn adequate and high ZnO (2500 mg/kg) supplemented diets based on the species level partial least square discriminant analysis [16].

Features of specific investigations are likely to influence the apparent effects of Zn supplementation on the composition of the microbial community and next generation sequencing can help identify a plethora of shifts that can be challenging to interpret. Moreover, it is generally recognized that significant variations in community structure may not equate to meaningful changes in its function, which in essence shapes interactions with the host. Metagenome functional potential analysis revealed that gene ontology (GO) terms ‘metal ion binding’, ‘metal ion transport’, ‘penicillin binding’, and ‘β-lactam catabolism’ had the highest abundance for pigs fed high ZnO (2500 mg/kg), indicating strategies related to managing the stress response and high Zn exposure, and the potential to reduce the abundance of GO terms for ‘carbohydrate metabolic process’, which could suggest reduced community metabolic and cross-feeding activity [16]. Interestingly, and in support of the functional analysis, high dietary ZnO reduced the concentration of the metabolites acetate, propionate, n-butyrate, total short chain fatty acids, and ammonium in colonic digesta. In another study, pharmacological Zn supplementation increased pentadecanoic acid (C15:0) and α-linolenic acid (C18:3) in pig feces, presumably via altered microbial activity, at 7 days post-weaning, following weaning at 21 days of age [22].

Regulatory mechanisms in the intestine strive to maintain homeostatic control. Feeding high levels of ZnO upregulates the Zn exporter, ZnT1, downregulates the importer, Zn importer 4, and reduces apparent ileal zinc digestibility, which resulted in higher total, free ion and protein-bound zinc in the colon of Landrace piglets at 3 weeks post-weaning, compared with those supplemented with lower dietary ZnO (40 or 110 mg/kg) or a chelated Zn source (Zn-Lysinate; 110 mg/kg) [16]. It remains somewhat unclear how the different zinc fractions in the GIT digesta interact with, and influence the microbiome. Various (mainly) negative, as well as positive, correlations between free ions or protein-bound zinc and numbers of different bacterial taxa or groups in the GIT were reported by [18], while seemingly similar Zn fractions between groups have been associated with different microbial profiles [16] or apparent fractional differences between groups associated with relatively similar gut microbiological parameters [20]. Excess Zn has been reported to be negatively correlated with the occurrence of enteric *E. coli* pathotypes, which could at least partially explain the associated reduction in diarrhea, but seemingly increases the carriage of virulence-associated genes within the intestinal *E. coli* populations [23], and has been proposed to slow the decline in fecal shedding of an administered *Salmonella* serovar [24].

Studies have reported that feeding supplemental Zn can alter the structure of the GIT. Recently, supra-nutritional levels of Zn, either standard or potentiated, increased the thickness of the gastric mucosa [22] or increased the villus height in the duodenum or jejunum of weaned pigs [19,25], which could promote the robustness of the GIT and/or nutrient’s digestion and absorption. In addition, high level standard or potentiated Zn reduced jejunal IL (interleukin)-8 and increased TGF (transforming growth factor)-β expression, or reduced the ileal concentration of IL-1β and increased the IL-1RA:IL-1 ratio, potentially indicating a lower inflammatory state of these tissues [22,25]. Moreover, gut-associated lymphoid tissues have been shown to express Zn transporters and T-cells to be activated or suppressed by feeding high dietary Zn for short (1–2 weeks) or longer (2–4 weeks) periods, respectively [26]. However, feeding a Zn amino acid complex (50 mg/kg (plus 75 mg/kg of zinc sulphate)) to 4-week old weaned pigs for 3 weeks prior to *Lawsonia intracellularis* infection increased T-cell numbers and the distribution of B-cells in the ileum, resulting in earlier seroconversion and fewer pigs with lesions, and of reduced severity [27]. In another recent study, there was no benefit from pre-feeding adequate or high dose Zn on in vitro epithelial barrier dysfunction, caused by alpha-haemolysin (HlyA)-expressing *E. coli*, of the colon from young pigs [28]. Additional luminal Zn (as Zn acetate) at challenge did, however, effectively prevent *E. coli*-induced epithelial barrier failure, which it was proposed was due to the luminal presence of free Zn ions. The pathological changes caused by the *E. coli*, including alterations of tight junction proteins claudin-4 and claudin-5, focal leak formation, and cell exfoliation, manifesting as reduced transepithelial electrical resistance (TEER), were absent from luminal Zn-administered colonic tissue. Other studies have reported effects of novel Zn sources, at low doses, on the expression of intestinal tight junctions, intestinal alkaline phosphatase, and TEER [20]. Moreover, Zn ions have been found to inhibit *Brachyspira hyodysenteriae* haemolysin biosynthesis in vitro [29] and ZnO at 2000 mg/kg feed (or higher) had a prophylactic effect against *B. hyodysenteriae* in a swine dysentery model in mice [30]. Recently, a Zn chelate has been shown to be effective against *B. hyodysenteriae* in swine [31,32].

Comparatively, there are fewer studies investigating the effects of Zn per se and/or from different sources on the gut microbiome of poultry, although there is growing interest. In young chickens, Zn deficiency reduced species richness in the caeca and increased UniFrac distances, suggesting lower diversity and relatedness of these bacterial communities compared to Zn replete birds [33]. In addition, Zn deficient chicks had expanded Proteobacteria, *Enterobacteriaceae*, and *Enterococcus*. As Zn is an essential mineral for many bacteria, the lower diversity and expansion of specific taxonomic groups indicates that those bacteria that can successfully compete for limited Zn or survive under Zn-limiting conditions are supported, but a reduction in diversity and expansion of the identified taxonomic groups have been associated with poorer gut health [34]. In a recent study, a Zn amino acid complex added to a wheat–rye based diet (at 60 mg/kg Zn) of broiler chickens increased Zn digestibility and villus length:crypt depth ratio, reduced abundance of some genera within the Proteobacteria phylum and oxidative stress, and improved starter phase feed conversion ratio compared to an equivalent amount of dietary Zn from Zn sulphate [35]. Additionally, in a necrotic enteritis broiler chicken challenge model, dietary Zn supplementation (90 mg/kg Zn) reduced NE lesions and mortality, as well as jejunal toll-like receptor 2 and ZnT5 expression [36]. Specifically, Zn proteinate reduced ileal *Lactobacillus* and counteracted jejunal upregulation of IL-8 and interferon-γ due to the challenge, and downregulated inducible nitric oxide synthase, in comparison to birds receiving the same amount of Zn from ZnSO_4_, indicative of attenuated jejunal inflammation. Two other recent studies found that dietary Zn (as Zn-glycine chelate) at 50 mg/kg increased ileal histological parameters and goblet cell count, antibody response to infectious bronchitis virus, serum paraoxonase (antioxidant) activity, and improved performance [37] or 30 and/or 60 mg/kg (as ZnSO_4_) increased intestinal morphological features and intraepithelial lymphocytes (IEL), of broiler chickens, the latter under heat stressed conditions [38]. Collectively, dietary Zn has the potential to modulate the gastrointestinal (GI)environment, notably under challenge conditions.

### 2.2. Copper in Pigs and Poultry

Similar to Zn, Cu is provided into the diets of pigs and poultry to meet their biological needs, and at higher inclusions for growth promotion, which is associated with improvements in gut health. Improved understanding of optimal dietary levels of Cu, and in what forms, to support health and performance, and minimize excretion, is essential. Recent work has indicated that Cu maybe absorbed somewhat more slowly from the pig intestine than Zn, particularly with increased levels of both minerals, which may allow greater Cu interaction with the GI environment [39].

In weaned pigs, Højberg et al. [23] reported that 175 mg/kg dietary Cu (from CuSO_4_) reduced lactic acid bacteria counts in the stomach, and coliforms in the cecum and the colon, while Namkung et al. [40] found the GI microbiota diversity to be reduced with 250 mg/kg additional Cu. A lower microbiota diversity is often considered undesirable, although often a feature of antimicrobial supplementation, but the reduction in the putative beneficial lactic acid bacteria was surprising. It was proposed that their suppression could improve nutrient availability to the host, while coliforms could encompass pathogens and thus their restraint would be favorable. Similarly, Mei et al. [41] reported reductions in caecum microbial diversity and the tendency for *Enterobacteriaceae* and lactobacilli to be decreased through increasing (to 250 mg/kg) dietary copper (from CuSO_4_) in weaned pigs.

More recently, using next generation sequencing, fecal α diversity indices were not affected by the dietary Cu level (6–300 mg/kg feed) in young pigs of a local Chinese breed (Suhuai) [42]. *Coprococcus*, *Roseburia*, and *Acidaminococcus*, notable butyrate-producing bacteria, were reduced by the higher level of dietary Cu, while *Streptococcus* and *Fibrobacter* were increased. It is again interesting that some of these abundance changes could be considered less desirable. Fecal metabolites indicative of protein and carbohydrate digestion, absorption, and metabolism were lower in the high Cu group, suggesting improved digestion of dietary nutrients. Pigs supplemented with a higher level of Cu also had lower serum tumor necrosis factor-α (TNF-α; inflammatory marker) and higher total antioxidant capacity. The correlation analysis identified potential relationships between the fecal microbiota and fecal metabolites or serum biochemical parameters, including some positive correlations between certain bacterial genera and serum TNF-α. In a previous study with rats fed different levels of dietary Cu, six fecal bacterial genera were significantly correlated with serum TNF-α [43]. Further work in weaned pigs revealed that up to 200 mg Cu/kg feed, as CuSO_4_, had no effect on α diversity indices of the ileal and caecal microbiota, with minimal or marked changes in the composition after shorter- (3 weeks) or longer-term (6 weeks) feeding, respectively, with differences in at least 40 genera across the ileum and caecum identified [44]. Moreover, pharmacological Cu increased *E. coli* richness in the hindgut and increased the frequency of multidrug resistant isolates. In addition, gut microbiota metabolic functions, such as energy metabolism, protein metabolism, and amino acid biosynthesis, were affected by Cu supplementation. Another recent study compared two Cu levels (15 and 160 mg/kg) from two sources (sulphate (SF) and hydroxychloride (HCl)) on the colonic microbiota of Large White × Landrace weaned pigs [45]. High Cu supplementation increased the relative abundance of *Methanosphaera* and *Roseburia*, and tended to reduce *Fibrobacter* and *Acidaminococcus*, highlighting some similarities and differences with the earlier work by Zhang et al. [42]. A comparison of high Cu from the two sources revealed that the HCl source reduced α diversity, the abundance of *Blautia*, *Streptococcus*, *Enterobacter*, *Fusobacterium*, *Escherichia*, *Propionibacterium*, *Mycobacterium*, *Desulfovibrio*, and increased *Pasturella*, *Methanobacterium*, *Mycoplasma*, *Bacillus*, and *Bulleidia*. Based on current perspectives, some of these modifications would be deemed beneficial (e.g., reduced *Escherichia*) and others less so (e.g., reduced diversity), which makes the observed changes intriguing. Recently, it was reported that pigs may freely choose diets with high Cu levels from different sources, it has been proposed that preference can be related to differences in Cu solubility [46]. However, prolonged feeding of high copper feeds to pigs may be detrimental as copper accumulates in host tissues and organs [47].

Copper has also been investigated for its effects on the GI environment of poultry. Studies have shown that dietary copper, as CuSO_4_ at 250 mg/kg [48] or tribasic CuCl (HCl) at 187.5 mg/kg [49], increased the similarity (coefficient) of the ileal mucosa-associated bacterial community of broiler chickens. In addition, 188 mg of Cu/kg feed from both sources influenced intestinal morphology and lamina propria and/or IEL, suggesting direct or indirect effects on gut physiology and immune responses [50]. Recently, a relatively low level of supplemental copper as nanoparticles (1.7 mg/kg) in the feed of broiler chickens reduced the abundance of caecal *Firmicutes* and increased *Bacteroidetes* [51]. At the genus level, *Lactobacillus* were decreased and *Bacteroides* increased. These shifts could be considered unfavorable (e.g., reduced *Lactobacillus*) but are somewhat consistent with those observed with growth-promoting antibiotics that are predominantly active against Gram-positive bacteria [52]. The use of Cu in the context of current industry challenges has also been studied. In an *Eimeria* challenge study, although growth performance was not improved, supplemental copper sulphate at 150 mg/kg reduced broiler chicken duodenal lesion score [53]. Notably, clear relationships between observed intestinal damage and growth performance are not always apparent [54]. In addition, *S. Typhimurium* colonization of caecal tonsils was significantly reduced, and unknown genus of *Lachnospiraceae* enriched in the caeca of *S. Typhimurium* infected broiler chickens supplemented with 250 mg/kg of Cu acetate [55]. *Lachnospiraceae* are key producers of short-chain fatty acids but have also been associated with intestinal diseases [56]. These studies highlight the potential of Cu to modulate the intestinal, particularly tissue-related, microbiota and immune-related features.

### 2.3. Manganese and Gut Health

Although essential for both host and microbial biological processes, there is a paucity of information regarding the influence of Mn on monogastric gut health. Human-focused studies found dietary Mn intake to be positively associated with fecal *Firmicutes* and negatively associated with fecal *Bacteroidetes* [57], and providing 15 mg/kg/day of MnCl_2_ to mice for 30 days increased gut transit time and altered the fecal metabolic profile [58]. Together, these data suggest that dietary Mn can modulate the gut microbiome and physiology, with the effect potentially sex specific [59]. Further work in mice reported that Mn deficiency compromised intestinal tight junctions, increased intestinal permeability, and aggravated dextran sulphate sodium (DSS)-induced colitis [60].

Work has also been conducted in livestock species. In dairy cows, Mn deficiency was associated with intestinal microbiota dysbiosis and poor recovery from botulism (caused by *Clostridium botulinum* neurotoxins) [61]. In lambs, 16 weeks feeding chelated Mn (Mn glycine chelate; 184 mg total Mn/kg DM) reduced the richness and diversity of the rumen *Eubacterium* population, while both the chelated and inorganic (Mn sulphate; 182.7 mg total Mn/kg DM) Mn sources influenced the carbohydrase activity of the rumen microbes [62]. Some recent studies have considered the impact of dietary Mn supplementation on broiler chicken responses to *Salmonella*. There was an early increase in *Salmonella* caecal content counts at 2 days post oral *Salmonella Typhimurium* inoculation of broiler chickens fed a higher dietary Mn (420 mg Mn/kg) level compared to those fed an adequate level (60 mg Mn/kg), suggesting that the higher Mn level initially supported their replication, but concurrently maintained jejunal villus height, which could be related to higher MnSOD activity and antioxidant capacity [63]. The higher Mn group also had higher percentages of CD4+ and CD8+ T-cells in peripheral blood and modulated cytokine gene expression in spleen, caecal tonsils, and bursa of Fabricius. In another *Salmonella Typhimurium* broiler chicken challenge study, greater duodenal villus height, increased jejunal *Lactobacillus* and *Bifidobacterium* and decreased *Salmonella* and *E. coli*, higher tight junction gene expression and reduced gut permeability, reduced numbers of *Salmonella* in both caecal content and spleen, greater splenic proinflammatory gene expression and mitochondrial MnSOD activity, and activation of acute-phase response, T helper type 1, and dendritic cell maturation pathways were associated with higher Mn supplementation (basal diet + 100 mg Mn/kg) in comparison to a Mn adequate (basal diet + 40 mg Mn/kg) and/or deficient (basal diet + 0 mg Mn/kg) diets [64]. Interestingly, there were also differences in specific broiler chicken peripheral blood T and innate cell proportions following *Salmonella Enteritidis* vaccination between birds fed diets providing 80 mg Mn/kg via MnSO_4_ supplementation alone or a 50:50 mix of MnSO_4_ and Mn amino acid complex [65], again indicating the importance of Mn source(s) for the biological response. Overall, whilst more work is needed is this area, Mn status and/or supplementation have the potential to influence the gut microbiome, barrier function, and immune responses.

**Table 2:** Mechanistic insights from recent livestock studies investigating copper, manganese, and zinc effects on the gut environment.

Mineral	Species	Mineral Supplementation Level and Source	Key Mechanistic Insights	Reference (s)
Zinc	Pig	Pharmacological (2500 mg/kg ZnO) Alternative Zn sources (50–220 mg/kg Zn)	Increased exporter and reduced importer expression. Higher ileal total, free ion, and protein-bound Zn. Changes in GIT microbiota composition and activity. Increased microbiome stress response and reduced carbohydrate processing. Lower intestinal inflammatory state. Increased Th1 response (to 2 weeks post-feeding) and then suppressed. Increased intestinal alkaline phosphatase expression and transepithelial electrical resistance (TEER). Changes in GIT microbiota—reduced *E. coli.* Increased T-cell numbers, earlier seroconversion and reduced lesions, and severity following *Lawsonia intracellularis* infection.	[16]
				[22]
				[26]
				[20]
				[27]
	Chicken	Zn deficiency Various forms (30–90 mg/kg Zn)	Reduced diversity and relatedness of caecal bacterial community. Reduced host oxidative stress and enhanced intestinal morphology. Reduced necrotic enteritis lesions, severity, and ileal lactobacilli, and altered jejunal inflammatory marker expression. Increased intestinal morphological features and IEL under heat stressed conditions.	[33]
				[35,37]
				[36]
				[38]
Copper	Pig	0–300 mg/kg (CuSO_4_ or HCl)	Reduction in notable fecal butyrate-producing bacteria, lower fecal protein and carbohydrate-related metabolites, and decreased serum TNF-α and higher total antioxidant capacity. Altered GIT microbiota, microbial energy and protein metabolism, increased *E. coli* and multidrug resistant isolates.	[42]
				[44]
				[45]
	Chicken	1.7–250 mg/kg (nanoparticles, CuSO_4_ or Cu acetate)	Modified caecal bacterial community. Decreased duodenal lesion score following mixed Eimeria challenge. Reduced *S. Typhimurium* colonization of caecal tonsils following oral challenge.	[51]
				[53]
				[55]
Manganese	Chicken	0–400 mg/kg (MnSO_4_)	Maintenance of jejunal villus height, higher percentage of CD4+ and CD8+ T-cells in peripheral blood, and modulated cytokine gene expression in spleen, caecal tonsils, and bursa of Fabricius following *S. Typhimurium* oral challenge. Modified jejunal bacterial community, reduced caecal Salmonella counts, increased duodenal villus height, tight junction gene expression and gut barrier function, activation of immune pathways, and greater splenic proinflammatory gene expression and mitochondrial MnSOD activity following *S. Typhimurium* oral challenge.	[63]
				[64]

### 2.4. Nanoforms

Nanoforms of Zn, Cu, and Mn have begun to be investigated more, academically, but, to our knowledge, these forms have so far only achieved very limited penetration in specific markets. Many of these studies have considered the effects of nanoforms outside the GI environment, as have been recently reviewed [66], although some investigations with such mineral forms have reported that the GI environment can be modified, often with much lower dietary levels, as outlined above (e.g., [19,51]), than have been used traditionally. Of course, some of the benefits reported systemically could have GIT-related origins and thus more studies focusing on the GI environment of pigs and poultry would be helpful. Nanoforms are suggested to have greater antimicrobial and immunomodulatory activity, increased bioavailability and/or less antagonistic interactions within the GIT, and, therefore, reduced environmental excretion than conventional forms [66,67], thus potentially offering a more sustainable approach to achieve the nutritional and supra-nutritional effects of Zn, Cu, and Mn.

## 3. Perspective and Conclusions

Zinc, Cu, and Mn are essential micronutrients for both the host and their colonizing microbes but can become toxic to cells when in excess. This creates an interesting dynamic as the host and microbiota compete for available micronutrients, with both having evolved elaborate strategies to seek an advantage. In a process referred to as ‘nutritional immunity’, the host’s immune-related proteins sequester metals to deprive bacteria (pathogens) of these essential nutrients [68]. In response, bacteria produce siderophores, molecules that bind metals with high affinity, to help in their acquisition of these nutrients. In addition, during infections, the host can target microbes with high levels of metals (e.g., copper) to use toxicity against them [69]. These elements have been used to meet the nutritional requirements of pigs and poultry and, particularly Cu and Zn, have been employed at supra-nutritional dietary levels to promote health and growth performance, with monogastric animals generally having a higher toxicity threshold than ruminants. The benefits of high levels of Cu and Zn, especially in pigs, for growth promotion and/or GIT ‘health’ are well established, but, due to concerns about high level excretion into the environment, appropriate dietary levels are being revised. In this review, by focusing on the latest research, we explore the most recent insights relating to supra-nutritional dietary levels of Cu, Mn, and Zn and the GIT environment, and, where possible, the use of alternative mineral forms seeking to maximize the associated benefits and minimize metal excretion (Table 2). Studies show that Zn, Cu or Mn can modify the GIT microbiota and/or (potential) pathogen populations. Some of these changes can be currently difficult to interpret and/or contrary to current beliefs about a desirable microbiota composition. The functional analysis of the microbiome is, therefore, becoming of increasing interest and has highlighted that supra-nutritional levels of these metals may, for example, increase stress response strategies and decrease the processing of carbohydrates, which is supported somewhat by changes in metabolites. Again, some of these modifications (e.g., carbohydrate processing) could seem counterintuitive (unless host digestion reduces availability) and require further investigation. Other studies suggest maintenance or enhancement of GIT structure and antioxidant capacity, improved tight junctions and barrier function, and activated or suppressed gut-associated immune responses (perhaps depending on the length of supplementation) from elevated feeding or status, of the selected minerals, as well as reduced susceptibility to pathogens. Notably, novel, alternative forms of the traditional mineral sources are reported to have similar or even superior, effects when providing significantly less of the mineral and closer to the nutritional requirement, which should drive reductions in excretion.

The benefits of supra-nutritional dietary levels of Zn, Cu, and Mn could be via direct effects on the gut microbiome and/or the host. One study indicated direct effects of free Zn ions in the gut lumen on pathogenic *E. coli*, while the relationships between total, free ion, and protein-bound zinc in the GIT and the microbiome are not entirely clear and warrant further investigation. However, the GIT is central to mineral absorption and excretion, and various interactions occur between, and as a result of, the presence of, for example, other minerals, dietary components, and GIT conditions. When minerals are fed in excess, their digestive efficiency decreases and thus minerals, perhaps in all forms, are presumably available throughout the GIT, and in higher concentrations, to (influence) the microbiota. In addition, characteristics of the mineral source will be important. Copper sulphate increased soluble Cu in the duodenal lumen, while Cu bioavailability increased throughout the GIT with tri-basic copper chloride (TBCC) [70], and the solubility of organic sources increases in weak acid conditions, which may slow dissolution and interactions that would reduce availability and/or activity [55]. However, purported increases in bioavailability, and thus lower dietary inclusions, of newer mineral products could put more emphasis on proximal GIT microbiome modification and/or host-mediated effects. Further research can unravel the fate and dynamics of ingested mineral forms and the relationships with host-mediated and/or microbiome-mediated effects throughout the GIT, which will allow these minerals to be provided more precisely to benefit the host and deprive pathobionts.

## 4. Conclusions

The nutritional requirements for Zn, Cu, and Mn are established and deficiency will impair health (including GI) and growth performance. The benefits of supra-nutritional or pharmacological dietary levels of these minerals for monogastric livestock are also clear but the mechanisms underpinning the effects are less so. However, novel mineral sources/products at relatively low dietary inclusions are reporting similar effects as supra-nutritional levels, and thus reaping the benefits while minimizing the environmental impact is possible.

## Data Availability

Not applicable.
